# Elements for successful implementation of a clinic-based health literacy intervention

**DOI:** 10.3389/fpubh.2022.977765

**Published:** 2022-10-28

**Authors:** Mark M. Macauda, Michelle A. Arent, Mayank Sakhuja, Brooks Yelton, Samuel Noblet, Delores Fedrick, Diana Zona, Cyndi New, William D. Isenhower, Abraham Wandersman, Daniela B. Friedman

**Affiliations:** ^1^Department of Health Promotion, Education, and Behavior, Arnold School of Public Health, University of South Carolina, Columbia, SC, United States; ^2^Center for Applied Research and Evaluation, Arnold School of Public Health, University of South Carolina, Columbia, SC, United States; ^3^Prevention Research Center, Arnold School of Public Health, University of South Carolina, Columbia, SC, United States; ^4^Chester County Literacy Council, Chester, SC, United States; ^5^South Carolina Hospital Association, Columbia, SC, United States; ^6^Self Regional Healthcare, Greenwood, SC, United States; ^7^Wandersman Center, Columbia, SC, United States

**Keywords:** patient-provider communication, health literacy, pragmatic intervention, clinic-based implementation, AskMe3, mixed methods

## Abstract

Improving health literacy is a national public health priority. Given the context of the COVID-19 pandemic, it is even more critical for health and medical information to be clear and understandable for patients and their families. Clinic-based programs to improve health literacy need to be pragmatic, feasible, and helpful for the implementing clinic and patients. This paper describes the development, implementation, and evaluation of a pragmatic, clinic-based health literacy intervention in a safety-net clinic that serves uninsured and indigent patients. Study methods are guided by a previous pilot study and components recommended for pragmatic interventions. An electronic readiness assessment was distributed to out-patient clinics affiliated with a statewide hospital association. The AskMe3 tool was used for the intervention as it is evidence informed and relatively easy to implement. Implementation included ongoing dialogue between the clinic and the academic research team. Within the implementing clinic, data collected from patients *via* verbally administered questionnaires was analyzed using descriptive statistics and chi-squares. Interview data collected from the clinic director was analyzed qualitatively for themes. The implementing clinic had some of the lowest average scores of the 34 clinics who participated in the initial readiness assessment. Despite this, they were able to successfully implement the health literacy intervention during a global pandemic. Eighty-eight participants completed patient questionnaires at this clinic. Most patients (96%) agreed the AskMe3 questions helped them talk with the doctor or nurse at their current appointment. Most (99%) also perceived the AskMe3 tool to be very helpful when used in a clinical setting. The clinic director offered that the staff initially thought the intervention would be difficult to implement. However, implementation by clinic volunteers with encouragement and prioritization of health literacy by the clinic director contributed to success. When considering interventions for clinical settings, a pragmatic approach can help with selection and implementation of a program that fits with the realities on the ground. Further, frequent technical assistance can help resolve implementation barriers. Interventions utilizing tools such as AskMe3, because of their simplicity, allow creative solutions to capacity issues for clinics who see a need for health literacy improvements.

## Introduction

Increasing health literacy of the United States (U.S.) population to improve public health is a national priority (1). Six Healthy People 2,030 objectives with a focus on health literacy have been proposed, including increasing the proportion of adults whose health care providers check their understanding of medical information and instructions, as well as decreasing the proportion of adults who report poor communication with their health care provider (2). In contrast to previous definitions of health literacy which have focused on an individual's ability to access, understand, and apply health information to inform decisions and actions (3), Healthy People 2030 emphasizes that health and medical organizations can and should play a significant role in enabling individuals to “find, understand, and use information and services to inform health-related decisions and actions for themselves and others” (2, 4). This intentional framing demonstrates clearly that the onus is not solely on the individual, and organizations charged with communicating medical information have a responsibility to address health literacy. While this is a change from previous iterations of Healthy People objectives, this is not a novel concept. Nutbeam's seminal work conceptualizing health literacy as a “personal asset” as opposed to a “clinical risk factor” established the critical role tailored health information, education, and communication can play in enhancing people's capacity to manage their health with the ultimate goal of improved health outcomes (5). An important aspect of this model was that clear, plain-language information could potentially raise people's awareness of social determinants of health and empower them to develop knowledge and skills that could improve a social determinant such as health literacy. These objectives underscore the need for interventions that can be implemented in a clinic setting to help improve health literacy.

Nationally, only 12% of U.S. adults have proficient health literacy (6). Limited health literacy coupled with health and medical-related information written at a reading level beyond the recommended Grade 5–6 level (7–11), presents a great risk to public health. Issues of complicated, technical information (9), poor understanding of information (12), and information lacking reference to potentially high-risk age or racial and ethnic groups (7–10) have become even more apparent during the COVID-19 pandemic, during which clear and timely communication is critical (12–14). Partnering on health messaging with community and volunteer organizations has been a successful strategy for creating and sharing more plain language and tailored health information with the public (13, 14), and focusing on organizational health literacy has been recommended as an effective method for improving health systems and patient outcomes, especially in the context of a pandemic like COVID-19 (15).

To find and implement interventions that are feasible in real world settings, work focused on improving organizational health communication and health literacy can utilize dissemination and implementation (D&I) principles and Glasgow and colleagues' five core values recommended for D&I science to guide programming and interventions (16, 17). These core values are: *rigor and relevance* (focusing on alternative research designs with high external validity, reflect real world clinical settings, and enroll diverse and underserved populations), *efficiency and speed* (using methods that rapidly inform decision making/implementation within health care practices, and use readily available or easily obtainable data), *collaboration* (partnering with communities/clinical settings using stakeholder-engaged approaches), *improved capacity* (making emerging methods and trainings available to researchers and stakeholder partners), and *cumulative knowledge* (referring to current and new resources about D&I science).

Layering these D&I core values with a pragmatic intervention approach yields context specific methods and results that are useful and meaningful to all stakeholders, clinicians, patients, investigators, partnering organizations, and funding entities (18–20). This is especially pertinent for clinic-based health literacy interventions that need to be feasible for the implementing organization and helpful for both clinic and patients. Four key components of pragmatic trials (21) are:

1. Approach–needing to focus on application within a specific context and ensuring usefulness by implementers and recipients, thus we asked patients to assess their own health literacy and the usefulness of the tool.
2. Model/Theory–needing to be simple and focus on context: which is why our health literacy work is guided by D&I's core values (17) and Nutbeam's health literacy asset model (5).
3. Design–needing to focus on organizations' resources and context to ensure results will be applicable, thus we conduct readiness assessments by Wandersman et al. (22) and Friedman et al. (23) prior to embarking on intervention work.
4. Measures–needing to be brief, broadly applicable, and sensitive to change, thus we used a team-based, iterative approach to development of all measures.

While community health literacy programs exist (14, 18, 19) with some being in clinical settings, (20, 23–26) there are a limited number of published intervention studies using the question-based, AskMe3 tool (27, 28) which is copyrighted and freely available from the Institute for Healthcare Improvement. None of these programs have been implemented in South Carolina, a state with the 13^th^ worst literacy rates in the country (23, 29). The purpose of this paper is to describe the development, implementation, and evaluation of a pragmatic clinic-based health literacy intervention that uses the evidence-informed AskMe3 tool, ongoing support and technical assistance, and a teach-back approach in a safety-net clinic in South Carolina serving uninsured and indigent patients.

## Methods

Study methods were guided by a previous pilot study with two hospital-associated clinics (23) and presented according to D&I core values and pragmatic trial components described earlier.

### Approach: Collaboration and cumulative knowledge

With pragmatic approaches in mind, AskMe3 was selected for this health literacy intervention as it is evidence informed, relatively easy to implement, and consists of existing materials in English, Spanish, and French from the Institute for Healthcare Improvement. Further, successful pilot testing of the health literacy intervention utilizing the AskMe3 tool was completed by a collaboration between research team and community partners (23). Recruitment of outpatient clinics for this intervention was accomplished through invitation emails sent *via* listservs by partners at a statewide hospital association and AccessHealth network.

### Measures: Collaborative development, rigor and relevance, efficiency, and speed

In order to gain an initial understanding of capacity to implement, as well as develop a base for further conversations, the project team of academic, clinical, and community partners developed an electronic readiness assessment guided by the Interactive Systems Framework (ISF) and the heuristic of Readiness = Motivation × General Capacity × Specific Capacity (R = MC^2^) (22, 23). The assessment comprised 60 items on a five-point scale (1 = strongly disagree; 5 = strongly agree) within the three constructs of motivation, general capacity, and innovation/intervention specific capacity. The individual assessment items were modified for this implementation (In addition, the readiness tool itself has been further revised since this implementation). Additional details about the collaborative development and testing of the tool are published elsewhere (23). An electronic link to the readiness assessment was included in the invitation email sent from partners to their respective listservs. Interested clinics from across the state completed the readiness assessments between November 2019 and the end of January 2020 before COVID-19 was designated a pandemic in the U.S. Thirty-four clinics completed the electronic readiness assessment.

After scoring the readiness assessment, the training coordinator conducted email outreach with all 34 clinics that completed the assessment. Due to COVID-19 restrictions, internal clinic flow restructuring, decreased staffing, and increased staff responsibilities; many clinics indicated a need to postpone their participation in the health literacy intervention.

Originally, we intended to follow up with clinics who had higher initial readiness scores and invite them to participate. As a result of these COVID-19 related changes, selection for participation in the intervention was extended to any clinic that had completed the readiness assessment and perceived they were able to begin. One safety-net clinic expressed interest in moving forward with the intervention protocol. The clinic has 13 paid staff members that come once per week, two full-time staff members (defined as 24 h per week in the clinic), six nurse practitioners (one full-time), and five volunteer medical doctors. The clinic mainly treats indigent patients ineligible for Medicare or Medicaid. The clinic serves as the medical home for these patients, many of whom have chronic conditions.

Since understanding clinic culture and practices was key to a successful intervention, the project training coordinator along with other members of the academic team conducted a virtual clinic visit *via* Zoom with the clinic director and his team, the purpose of which was to describe the general features of the intervention, review the readiness results, learn more about the clinic flow and management, and determine feasibility of implementation. The virtual visit protocol is presented in Supplementary File VirtualClinicVisit.doc. In preparation for the visit, an online survey was sent to the clinic that asked about staffing and clinic operations (such as hours, appointment length, and appointment tracking systems used), to help the training coordinator discuss workflow with the clinic.

### Design: Rigor and relevance, efficiency and speed, and improved capacity

The intervention was a one-group, pre-post design previously pilot tested at two clinics associated with the hospital of one of our clinical partners. Pre-post intervention measures were also developed, piloted, and modified based on the pilot implementation experience at these clinics (23). The academic team mailed all intervention-related materials to the clinic described in this paper, including the written protocol that was also explained verbally by the training coordinator, pre- and post-data collection instruments, and the intervention materials–AskMe3 pamphlets for volunteers to review with patients as well as posters for clinics to hang in the waiting room and patient examination rooms. A list of plain language medical terms compiled by the Institute for Healthcare Improvement was also shared with clinic staff to encourage them to communicate clearly with patients. The training coordinator set up weekly check-in calls with the clinic director developing a partnership with the clinic and provided training and technical assistance on implementation and data collection (such as discussing clinic workflow issues, working through implementation protocols, and answering questions about AskMe3 implementation).

As part of the protocol, the clinic's care team (implementers) wrote down responses as patients answered pre-post intervention questions during patient encounters. Pre-post intervention questionnaires were developed for this project. Completed documents were scanned and emailed through a secure email system, and the training coordinator entered all data into Qualtrics (30). Patients were administered the questionnaire (verbally) by clinic implementers three times across two clinic visits. The first patient questionnaire was completed before the patient saw the healthcare provider and included the single item literacy screener (SILS) (31), “*How often do you need to have someone help you when you read instructions, pamphlets, or other written material from your doctor or pharmacy?”* Using a four-level Likert scale (extremely comfortable, somewhat comfortable, slightly uncomfortable, very uncomfortable), patients were also asked in general, how comfortable they were in asking questions during clinic/doctor's visits, and if they normally understood what they need to do when they leave the clinic (yes, no, sometimes). The second patient questionnaire was administered by a volunteer during the same visit after the patient saw the healthcare provider. Questions focused on the patient's understanding of the information they received during the current office visit, their level of comfort asking questions in this visit, if they felt AskMe3 materials helped them to talk to the healthcare staff, if AskMe3 materials helped them to understand what they need to do when they leave the clinic, as well as their opinion as to the helpfulness of AskMe3 in a clinic type setting. Patients also answered questions about the communication methods of the healthcare provider including if they used words that were easy to understand, real world examples to aid in understanding, and/or pictures, charts, or drawings to explain information. Additionally, patients were asked about their involvement in decision making and being given a chance to ask all the medical questions they had.

The third patient questionnaire was completed at the patient's next visit to this clinic and asked the same SILS question, along with questions about any hospitalizations or emergency room visits since their last clinic visit and if the patient had used AskMe3 in other settings since their last visit. Following intervention completion, the first author conducted a one on one interview with the clinic director *via* Zoom, to gain his perspective on the intervention, how it was implemented, and successes and barriers.

### Data analysis

#### Readiness assessment across multiple clinics

Responses to the readiness assessment and clinic patient questionnaires were imported into SPSS 26.0 (IBM Corp, Armonk, NY, USA) for descriptive analysis (frequencies, means, and percentages). Readiness assessment scores for the three domains (motivation, general capacity, innovation specific capacity), and overall readiness scores were calculated for each of the 34 clinics. Prior to score calculation, some item scales were transposed, so that in all cases, higher numeric scores equaled a greater degree of readiness. These scores were then averaged across clinics. Next the difference between our case study clinic individual score and the overall mean score across clinics was calculated.

#### Instruments used at the implementing clinic

Responses to questions within each patient questionnaire (see Supplementary material) were analyzed using frequencies and crosstabulation with chi-square to check for statistical significance where appropriate (*p* < 0.05).

#### Interview with director of the implementing clinic

The interview was recorded and the first author, who has a PhD in medical anthropology, (who served as interviewer) reviewed the Zoom interview recording for themes and explanations around readiness and implementation using a grounded theory approach (32). Verbatim quotes were compiled to validate thematic coding and interpretation. The interview lasted 48 Min (see Supplementary material).

## Results

### Readiness assessment across multiple clinics

The mean motivation score for the implementing clinic (M_IC_) was 2.94 while mean motivation score across all 34 clinics (M_AC_) was 3.91, with standard deviation (SD) of 0.65. For the section on motivating factors, the three items with the greatest difference between the implementing clinic and the mean for all clinics were: “Improving patients health literacy is a top priority for our clinic” (Priority Subcomponent: M_IC_, 2 vs. M_AC_, 4.4), “We already see how the AskMe3 program could have benefits for our patients” (Observability Subcomponent-: M_IC_, 2 vs. M_AC_, 3.6), and “Overall, the AskMe3 program sounds difficult to implement” (Complexity Subcomponent: M_IC_, 2 vs. M_AC_, 3.5).

With respect to the innovation specific capacity score, the M_IC_ was 2.67 vs. all clinic mean 3.47, SD 0.63. The three items with the greatest difference between implementing clinic mean and the all-clinic mean were: “We have the knowledge and skills to implement the AskMe3 program.” (Innovation-specific knowledge, skills, and abilities Subcomponent: M_IC_, 2 vs. M_AC_, 3.7), “We have a patient and family advisory council or advocate that may partner with us on the AskMe3 program.” (Interorganizational relationship Subcomponent: M_IC_, 1 vs. M_AC_, 2.7), and “We are interested in receiving support (e.g., training, technical assistance, etc.) for our clinic's participation in the AskMe3 program.” (Innovation-specific knowledge, skills, and abilities Subcomponent: M_IC_, 3 vs. M_AC_, 4.3).

For the general capacity score, the implementing clinic had a mean of 3.36 compared with the all-clinic mean of 4.04, SD 0.56. The three items with the greatest variation between implementing clinic and overall mean were “We have diverse sources of revenue and resources (e.g., multiple grants).” (Resource utilization Subcomponent: M_IC_, 2 vs. M_AC_, 3.8), “Staffing levels are adequate to carry out collaborative health system activities.” (Staffing Subcomponent: M_IC_, 2 vs. M_AC_, 3.6) and “Our clinic consistently implements programs that are aligned with our clinic's mission and strategic plan.” (Culture Subcomponent: M_IC_, 3 vs. M_AC_, 4.4).

### The clinic's readiness to implement from the clinic director's perspective

While their responses indicated they perceived challenges to their implementation readiness, the clinic staff (and the director in particular) expressed a strong desire to participate in the health literacy intervention. The clinic director felt as though the subject matter (improving health literacy) addressed an ongoing problem and was an important focus area for patient interaction.

*It's a long going issue with our patients, our demographic of patients; a lot of them are educationally challenged, and we just noticed through our time here*, that *dealing with the nurses and the providers, a lot of them basically didn't know even the most fundamental questions to ask of them*.

The clinic director also felt motivated to support the University.

*And thoroughly, it's my alma mater, and I have to be a good Carolinian and help my alma mater if they need help*.

When asked about why the clinic perceived challenges to their readiness, the clinic director reported clinic personnel did not fully understand what the implementation of AskMe3 would entail and visualized it as a potential burden. The director responded to staff concerns by putting the intervention into context, underscoring how the program related to literacy issues staff members had seen in the clinic, as well as framing it as a chance to make a larger contribution.

*At first they were a little apprehensive, like gosh, this is just something else we have to do, particularly nursing service but I told them, remember all along we have had this discussion about these folks coming in, and not knowing what questions to ask, and they are leaving us and we wonder if they really understood what we were asking them, so this, again, this is a chance to be something bigger than us*. that *we can help on a global, a statewide basis*.

At the time of the initial readiness assessment, the clinic did not consider its volunteers, who assisted with patient navigation and clinic workflow, as a potential part of clinic capacity. The clinic director explained that since the details of the intervention were not fully known, the clinic staff did not know whether volunteers had the capacity to implement. However, during the virtual site visit and subsequent conversations as the training coordinator and director discussed the readiness assessment scores, clinic workflow, and the details of the implementation, it was determined the intervention would be feasible, and volunteers could deliver it.

*Well I didn't really realize at the time* that *we would have the human resources to do it, and didn't realize at* that *time* that *our volunteers would be such an integral part, and would be so willing to help. But once. I was wrong, and I am glad I was wrong, but our volunteer really stepped up*.

*I didn't know if they would be the right people to do it. But after talking to them and realizing they did have the skill set to do this, then they were a natural fit*.

### Intervention implementation findings

#### Intervention participants from the implementing clinic

A total of 88 participants from the implementing clinic completed patient questionnaires at all three timepoints. Mean age of all participants was 51.58 years with an age range of 23–64 years. Slightly less than two-thirds (62.5%) of the participants were male. More than half (53.5%) of the participants were identified as Black or African American, 45.5% were identified as White or Caucasian, and one participant was identified as Mexican. Twenty (22.7%) participants indicated less than high school education, 21.6% had some college, technical or vocational training, 42% were high school graduates, 11.4% had an associate degree, and 2.3% had a bachelor's degree. None of the participants had any type of health insurance coverage.

#### First patient encounter (questionnaire 1 and introduction of AskMe3)

About 81% of the participants reported they rarely or never needed any help for reading instructions, pamphlets, or other written materials from doctors or pharmacies. Most (83%) reported comfortability in asking questions during the doctor's visit as well as (90.9%) understood what they needed to do after doctor's appointment. Only 2.3% of participants reported being very uncomfortable in asking questions during doctor's appointment.

#### Second patient encounter (questionnaire 2)

Following their appointment with the healthcare provider at the clinic, all participants reported being comfortable asking questions. All participants reported providers used easy-to-understand language and explained things in the simplest ways for patient's better understanding. More than 95% of the patients were given a chance to ask questions during the appointment and were involved in decisions about their health. When asked if the AskMe3 questions helped them to talk with the doctor or nurse at this current appointment, the majority (90.9%) agreed. The AskMe3 questions helped 95.5% of the participants understand what they needed to do after leaving the clinic. The AskMe3 tool was perceived to be very helpful when used in a clinical setting among almost all the participants.

When asked why participants felt AskMe3 would be a helpful tool, they reported several reasons, such as AskMe3's wide applicability, potential in improving and simplifying patient care and patient-provider communication, helpful in better understanding of problem, treatment plan, and its benefits. Some also felt the AskMe3 tool made them comfortable, empowered, and enabled them to take care of their own health. When asked if they could see themselves using the AskMe3 tool elsewhere, participants reported the potential of its use in diagnostic services (*n* = 14), pharmacies (*n* = 14), emergency medical situations (*n* = 2), specialty care (*n* = 3), and other service sectors such as auto service (*n* = 4).

#### Follow-up patient encounter (questionnaire 3)

A patient's follow-up appointment was on average between 7 and 8 weeks later. At this appointment, the number of patients that reported they never needed to have someone help them reading instructions, pamphlets, or other written materials at doctors' clinic or pharmacies significantly increased from 50 (56.8%) to 71 (80.7%). Twenty (22.7%) (chi-square value = 39.13, *p* = 0.001) participants reported they had used AskMe3 questions since their last clinic visit. Pharmacies (*n* = 7), laboratories or diagnostic services (*n* = 2), other clinic and hospitals (*n* = 4) were the reported settings where patients had used AskMe3 questions since their last visit. Some patients noted they had not used AskMe3 in any other setting simply because they had not gone anywhere due to COVID-19 restrictions.

#### Intervention results from the clinic director's perspective

During the interview with the clinic director, he lent some insight into the results of the patient questionnaires, as well as some overall insights about the intervention. The clinic director explained that implementers framed the intervention to patients as an initiative to assist the university with a study on how to improve patient-provider communication. This helped avoid insulting or offending patients with literacy issues.

*You can't be condescending, to people, you have to, not say* that *we know you are uneducated and need this help, there are certain interpersonal skills* that *go along with this, to say to a patient coming in, “Mr. Jackson, we would like, we need your help in trying to educate the people of South Carolina, about the proper questions to ask, would you yourself like to participate in this, this is a chance for you to be part of something bigger, and you may also benefit from this”. …A lot of our people have never had* that *opportunity*.

The clinic director also said he felt patients over-estimated their health literacy skills. For example, some of the clinic diabetic patients confused blood sugar with blood pressure.

*Compliance is a big problem with our patients, and a lot of the compliance issues are due to, I think, literacy deficits. I think they grossly overestimated their ability to ask the right questions*.

Overall, study participation took seven minutes, including administration of the questionnaires. The actual intervention using AskMe3 took considerably less time.

## Discussion

Of the 34 participating clinics, the implementing clinic described in this paper had some of the lowest average initial readiness scores. However, they recognized literacy as a challenge for their patient population, and the director was motivated to address the issue. The strategy of using the readiness assessment, as well as additional survey information on clinic flow, as the groundwork for a discussion on implementation options allowed this clinic to identify a path forward. Perceived implementation barriers were re-evaluated through Zoom and telephone discussions once the clinic learned more about the intervention steps. Despite the initial overestimation of complexity and assumed limited staff capacity, the clinic director gained buy-in from healthcare providers and office staff and volunteers. The clinic director's ability to communicate the importance of health literacy, the ways in which the intervention would benefit them and the clinic, as well as the patients and community at large served to move the implementation forward. Additionally, the clinic director and staff members liked the idea of supporting efforts of a local university which many clinic staff attended. The clinic director's leadership, direction, and support enabled staff members' successful implementation of this intervention- making him the program/intervention champion (33). The clinic leadership structure likely allowed the director considerable latitude in implementation. This may not have been as successful in a private practice without buy-in from partners who would have greater decision-making authority concerning implementation of new initiatives. Ultimately the clinic, with the support of the research team, was able to successfully implement the health literacy intervention with limited staff and during a global pandemic.

Implementation was further facilitated by the ease with which the intervention fit in with the established clinic workflow. This is a testament to the selection of a pragmatic intervention developed using D&I core values and pragmatic trials (21, 34, 35). D&I core values focused on ensuring the intervention was relevant to a real-world clinical setting, establishing partnerships with clinics and implementers through clear and frequent communication and support, providing appropriate training and technical assistance for ensuring the intervention would fit within the clinic flow and take minimal time and effort, and sharing resources about AskMe3 and implementation strategies increased capacity of this clinic to focus on health literacy and implement this specific type of pragmatic intervention. Use of AskMe3 in other clinical settings has also resulted in positive outcomes. For example, a study that incorporated AskMe3 questions into a video for patients with post-myocardial infarction showed significant improvements in knowledge among the intervention group (36). Furthermore, parents at a Hispanic pediatric clinic who were provided with AskMe3 materials demonstrated increased awareness and use of the questions within 6 months (27).

Strengths of our intervention protocol were that it was evidence-informed using AskMe3, and we ensured the data collection for implementation fit within the flow of the clinic (design). The data collection instruments were also brief, and pilot tested prior to implementation. The instruments and implementation protocol were discussed with the clinic director and volunteers to ensure they would be applicable to their patient population (measures).

At the one implementing clinic, most patients rated their own health literacy as high, and their need for assistance low. This may be because individuals overestimate their health literacy when asked for a self-assessment (37). Despite this initial assessment, clinic patients did feel the AskMe3 brochure was helpful, and they were more confident in their abilities to understand relevant information post intervention. The patients' view of their literacy underscores the importance of being aware of patient perspectives when planning and framing interventions. Exploring health literacy as a personal asset and how improved patient-provider communication could help patients understand and take control of their health was an important feature for the research team and clinic implementers (model).

This study has limitations. First, while we described the intervention on the readiness assessment survey and provided a link to the pamphlet, we did not provide an in-depth study protocol, nor spoke to the potential time commitments of AskMe3 or suggested how it might integrate into clinic workflow. We shared this with the clinic director during the follow-up virtual visit. This may have affected the clinic's scoring on the assessment, specifically regarding the innovation-specific capacity component. Second, this analysis focused on one clinic, and we cannot generalize our findings to other clinics or to other health literacy interventions. Further, many patients had not yet used the AskMe3 tool in other medical settings given the context of COVID-19. However, they did express the AskMe3 tool would be helpful in other healthcare settings, such as pharmacies. Despite these limitations, overall, the intervention, including handwriting patient responses to questions and sharing he AskMe3 materials, took under 10 min of clinic staff or volunteer time, and they found it relatively simple to implement.

Our readiness assessment occurred before the beginning of the COVID-19 pandemic; however, the intervention was begun during the pandemic. A re-assessment, perhaps using a specifically designed readiness tool (38), may have helped us adapt the implementation for the context of the pandemic. However, our ongoing dialogue with the clinic allowed us to navigate any disruptions of clinic processes or capacities that may have occurred due to the pandemic.

## Conclusion

When considering an intervention to be used in a clinical setting, a pragmatic approach to implementation, combined with ongoing technical assistance, can help prepare research teams, partners, and clinics to select and implement a program that fits well with the realities on the ground.

Interventions utilizing tools such as AskMe3, because of their simplicity, allow creative solutions to capacity issues for clinics who see a need for health literacy improvements, but cannot spare clinician time. For assessments and implementations to be successful, it is important to thoughtfully review the capacity needed to implement and engage in ongoing dialogue about implementation. This avoids clinic underestimation of capacity and ability, resulting in missed opportunities and overlooked creative solutions. Further, patients' views and assessments are key pieces of information, and it is important to consider their perceptions when framing the intervention. In this case given the relative ease of implementation, and the minimal resources needed, even a small improvement in health literacy for patients makes a compelling case for its use.

## Data availability statement

The original contributions presented in the study are included in the article/Supplementary material, further inquiries can be directed to the corresponding author.

## Author contributions

MM, DFr, and MA led the conceptualization and design of the study and prepared the initial outline of the manuscript. MM served as lead author, led qualitative analysis, and contributed to results, discussion, and conclusions. MA was instrumental in the write-up of the methods and in review of all other manuscript sections. MS led the analysis of and write-up of quantitative data. BY and SN contributed to the discussion and conclusions. AW, DZ, DFe, CN, and WI reviewed the full drafts of the paper. All authors read and approved the final manuscript.

## Funding

This publication was supported by the Centers for Disease Control and Prevention of the U.S. Department of Health and Human Services (HHS) as part of a financial assistance award (U48 DP006401) and The Duke Endowment (6816-SP).

## Conflict of interest

Author DFe was employed by Chester County Literacy Council. The remaining authors declare that the research was conducted in the absence of any commercial or financial relationships that could be construed as a potential conflict of interest.

## Publisher's note

All claims expressed in this article are solely those of the authors and do not necessarily represent those of their affiliated organizations, or those of the publisher, the editors and the reviewers. Any product that may be evaluated in this article, or claim that may be made by its manufacturer, is not guaranteed or endorsed by the publisher.

## Author disclaimer

The contents are those of the authors and do not necessarily represent the official views of, nor an endorsement, by CDC/HHS, or the U.S. Government, or The Duke Endowment.

## Abbreviations

D&I: Dissemination and Implementation
ISF: Interactive Systems Framework
R=MC^2^: Heuristic of Readiness = Motivation x General Capacity x Specific Capacity
SD: Standard Deviation
M_IC_: Mean Implementation Clinic
M_AC_: Mean All Clinics
U.S.: United States.
