# Supplementary material for: Elements for successful implementation of a clinic-based health literacy intervention
Source: Front Public Health. 2022 Oct 28;10:977765. doi: 10.3389/fpubh.2022.977765 (PMC9650509; doi:10.3389/fpubh.2022.977765)
Supplement: Supplementary file 1 [file Data_Sheet_1.docx]

**First Patient Encounter**

**Appt Date: _____________ Pt ID#: ____________**

**Please note the following prior to starting the interview:**

|  | Yes | No |
| --- | --- | --- |
| Is patient alone? |  |  |
| Is translator needed? |  |  |
| Translator in person? |  |  |
| Translator on phone? |  |  |

**Please read each question to the patient and mark their response(s) before the physician arrives. Please submit completed survey (Qualtrics) or return survey to designated location for data entry (paper).**

1. How often do you need to have someone help you when you read instructions, pamphlets, or other written materials from your doctors or pharmacies?

☐ Always*

☐ Often*

☐ Sometimes*

☐ Rarely

☐ Never

***If answered above with Always, Often, Sometimes, please ask:**

1a. Who typically helps you with this?

2. In general, how comfortable do you feel asking questions during your clinic/doctor’s visit?

☐ Extremely comfortable

☐ Somewhat comfortable

☐ Slightly uncomfortable

☐ Very uncomfortable

3. In general, do you normally understand what you need to do when you leave the clinic/doctor’s office?

☐ Yes

☐ No

☐ Sometimes

**4. Discussion about Ask Me 3 Brochure** (Care team member will provide information about Ask Me 3 and provide brochure to patient about the Ask Me 3 questions and how to use them): **(See Protocol Document)**

Do you have the paper/brochure the receptionist gave you?

Great, could you go ahead and take it out? Let’s take a look at it.

It’s something to help you get the most out of your appointment.

These are 3 questions you can ask here, or at any doctor’s office, or pharmacy- anywhere- to be sure you have the information you need about your health.

What’s my main problem?

What do I need to do?

Why is it important for me to do this?

We want you to have the answers to these questions so that you can take care of your health.

Sometimes you may get a lot of information at a doctor’s appointment. It may be confusing, and we may not be clear in what we mean. Asking these 3 questions helps to make sure everyone is on the same page. It helps us remember to be clear with the information we share with you and it helps you ask any questions you may have about what we shared. Remember, the goal of your visit is for you to have a clear plan of what to do when you leave here.

If you ask these questions and we still haven’t explained it in a way that is easy to understand, we want you to ask us again. Is that something that you would feel comfortable doing?

Ok, great!

This brochure has places to mark what your main problem is today, and what the doctor wants you to do about it (to take care of your health). There are also places to make notes so you’ll have the information you need when you leave here today.

Please select the version of brochure patient was given:

☐ AskMe3 original writing
☐ Emoji and picture

5. Before we wrap up I just have a couple of questions about you:

- 1. Could you tell me your age: ___________
  2. How far did you go in school? ________________________

☐ Less than high school
☐ High school graduate or GED
☐ Some college, technical or vocational training
☐ 2 year or Associate degree
☐ Bachelor’s degree
☐ Advanced/graduate degree
☐ Doctorate

The following table should be completed by the care team member based on the clinic’s patient data.

| **Patient data to be collected from Clinic** | **Enter responses in each row** |
| --- | --- |
| Sex |  |
| Age |  |
| Race |  |
| Ethnicity |  |
| Primary Language |  |
| Zip Code |  |
| Primary Diagnostic Code(s) |  |
| Health Insurance Type |  |
| Referring Physician |  |
| Date of Last Hospitalization |  |
| Current Prescriptions |  |

**Second Patient Encounter**

**Appt Date: _____________ Pt ID#: ____________**

**Please read each question to the patient and mark their answer(s). Please submit completed form (Qualtrics) or return to designated location for data entry (paper)**

**In Exam Room**

1. The following questions are about today’s visit in this office. When you spoke with doctors, nurses, or other health providers in this office:

|  | Yes | No | Don’t remember/NA |
| --- | --- | --- | --- |
| Did they use words that were easy to understand? |  |  |  |
| Did they use real world examples to help you understand what they were saying? |  |  |  |
| Did they explain things in a way that you could understand? |  |  |  |
| Did they use pictures, charts, or drawings to explain the information they were giving you? |  |  |  |
| Did they involve you in decisions about your health as much as you wanted? |  |  |  |
| Did they give you a chance to ask all of the medical questions that you had? |  |  |  |

1. How comfortable did you feel asking questions during your clinic/doctor’s visit today?

☐ Extremely comfortable

☐ Somewhat comfortable

☐ Slightly uncomfortable

☐ Very uncomfortable

1. Did the AskMe3^TM^ questions help you to talk with the doctor/nurse today?

☐ Yes

☐ No

☐ Maybe

☐ I don’t know

1. Did the AskMe3^TM^ questions help you to understand what you need to do when you leave the clinic?

☐ Yes**

☐ No*

☐ Maybe*

☐ I don’t know*

**If YES is selected, ask patient to repeat back what they need to do: directions/medications/follow up visit

*If NO/MAYBE/I DON’T KNOW is selected, revisit discharge directions/medications/follow up visit and ask patient to repeat back.

5a. Do you feel that the AskMe3^TM^ questions would be a helpful tool to use in a clinic setting like this one?

☐ Yes

☐ No

5b. Why or why not?

6a. Could you see yourself using the AskMe3^TM^ questions somewhere else?

☐ Yes

☐ No

6b. If so, where? (give prompts like pharmacy, specialty care, diagnostic test appointments, etc.)

**Follow-up Patient Encounter**

**Appt date: ____________________ Pt ID#: _________________**

Follow-up conducted at patient’s next visit (~4-6 weeks later) or over the phone (same time frame

1. How often do you need to have someone help you when you read instructions, pamphlets, or other written materials from your doctors or pharmacies?

☐ Always*

☐ Often*

☐ Sometimes*

☐ Rarely

☐ Never

***If answered above with Always, Often, Sometimes, please ask:**

1a. Who typically helps you with this?

2. Have you had any emergency room visits since your last clinic visit (insert date________)?

☐ Yes

☐ No

If so, what was it related to?

3. Have you been hospitalized since your last clinic visit (insert date ________)?

☐ Yes

☐ No

If so, what was it related to?

4. Have you used AskMe3^TM^ questions in any setting (pharmacy, specialty care, medical testing etc) since your last clinic visit?

☐ Yes

☐ No

If so, what was it related to?

**Virtual Clinical Visit Protocol**

1. Welcome and introductions
2. Discuss purpose of the virtual clinic visit, importance of improving health literacy and benefits to patient and clinic
3. Explain intervention protocol
4. Gain understanding of clinic flow for best fit of intervention protocol
5. Determine/discuss clinic champion’s role
6. Determine care team/clinic staff members’ roles in implementation
7. Discuss data collection and reporting
8. Determine potential start date and follow-up timeline
9. Address/answer any questions from clinic staff
10. Following the virtual visit, send follow-up thank-you email with contact information and any additional requested documents or information about the initiative

**Interview Guide with Clinic Director of the Implementing Clinic**

1. Think back to when you were first filling out the readiness assessment:
   1. What was your clinic’s feelings about trying a health intervention
   2. How did that reflect in the readiness assessment
2. Ultimately, you decided to try to implement AskMe3 despite your low perceived readiness, what led to that decision?
3. Your main strategy was to use volunteers to help you administer AskMe3.  How did that decision come about?
4. Can you tell me a little about how you worked the volunteers into your workflow
   1. Were there any barriers to doing this?
5. How did the staff and providers receive the intervention
6. How do you think this has all worked out?
